# Pulmonary hypoplasia and anasarca syndrome in Cika cattle

**DOI:** 10.1186/s13028-016-0220-9

**Published:** 2016-06-06

**Authors:** Tanja Švara, Vasilij Cociancich, Katarina Šest, Mitja Gombač, Tomislav Paller, Jože Starič, Cord Drögemüller

**Affiliations:** 1 Institute of Pathology, Forensic and Administrative Veterinary Medicine, Veterinary Faculty, University of Ljubljana, Ljubljana, Slovenia; 2 National Veterinary Institute, Veterinary Faculty, University of Ljubljana, Ljubljana, Slovenia; 3 Clinic for Ruminants, Veterinary Faculty, University of Ljubljana, Ljubljana, Slovenia; 4 Institute of Genetics, Vetsuisse Faculty, University of Bern, Bern, Switzerland

**Keywords:** Pulmonary hypoplasia, Anasarca, Lymphatic system, Hydrops foetalis, Rare disease

## Abstract

**Background:**

Hydrops foetalis is defined as excessive fluid accumulation within the foetal extravascular compartments and body cavities. It has been described in human and veterinary medicine, but despite several descriptive studies its aetiology is still not fully clarified. Pulmonary hypoplasia and anasarca (PHA) syndrome is a rare congenital abnormality in cattle that is characterised by hydrops foetalis including extreme subcutaneous oedema (anasarca) and undeveloped or poorly formed lungs (pulmonary hypoplasia). Until now, sporadic cases of PHA were reported in cattle breeds like Australian Dexter, Belted Galloway, Maine-Anjou, and Shorthorn. This report describes the first known cases of PHA syndrome in Slovenian Cika cattle.

**Case presentation:**

A 13-year-old cow aborted a male calf in the seventh month of pregnancy, while a male calf was delivered by caesarean section on the due date from a 14-year-old cow. The pedigree analysis showed that the calves were sired by the same bull, the dams were paternal half-sisters and the second calf was the product of a dam-son mating. Gross lesions were similar in both cases and characterized by severe anasarca, hydrothorax, hydropericardium, ascites, hypoplastic lungs, absence of lymph nodes, and an enlarged heart. The first calf was also athymic. Histopathology of the second affected calf confirmed severe oedema of the subcutis and interstitium of the organs, and pulmonary hypoplasia. The lymph vessels in the subcutis and other organs were severely dilated. Histopathology of the second calf revealed also lack of bronchus associated lymphoid tissue and adrenal gland hypoplasia.

**Conclusions:**

The findings were consistent with known forms of the bovine PHA syndrome. This is the first report of the PHA syndrome occurring in the local endangered breed of Cika cattle. Observed inbreeding practice supports that this lethal defect most likely follows an autosomal recessive mode of inheritance. In the light of the disease phenotype it is assumed that a mutation causing an impaired development of lymph vessels is responsible for the hydrops foetalis associated malformations in bovine PHA.

## Background

Hydrops foetalis (HF) is defined as excessive fluid accumulation within the foetal extravascular compartments and body cavities, characterized by generalized skin thickness, placental enlargement, pericardial or pleural effusion or ascites [1, 2]. Nonimmune HF is a feature of many genetic disorders e.g. related to alpha-thalassemia (OMIM 236750). HF has been described in human (OMIM 613124) and veterinary medicine (OMIA 000493), but despite several in-depth studies its aetiology remains unclear [2].

Pulmonary hypoplasia and anasarca (PHA) syndrome is a lethal genetic defect of cattle that is characterised by HF with extreme subcutaneous oedema (anasarca) and undeveloped or poorly formed lungs (pulmonary hypoplasia) [3–6]. The rarely occurring bovine diseases HF (OMIA 000493–9913) and PHA (OMIA 001562–9913) were both reported to be recessively inherited in Australian Dexter, Belted Galloway, Maine-Anjou, and Shorthorn cattle [3–6]. Lesions similar to those found in PHA affected cattle have also been described in a sheep (OMIA 000493–9940). Sporadic cases of Cheviot and Cheviot-Texel cross-lambs showed HF [7] and in Spanish sheep an autosomal recessive inherited form of HF was observed [8].

This report describes two cases of the PHA syndrome in Cika cattle, representing a previously unnoticed most likely recessively inherited genetic defect in this autochthonous breed from Slovenia [9].

## Case presentation

**Fig. 1:**
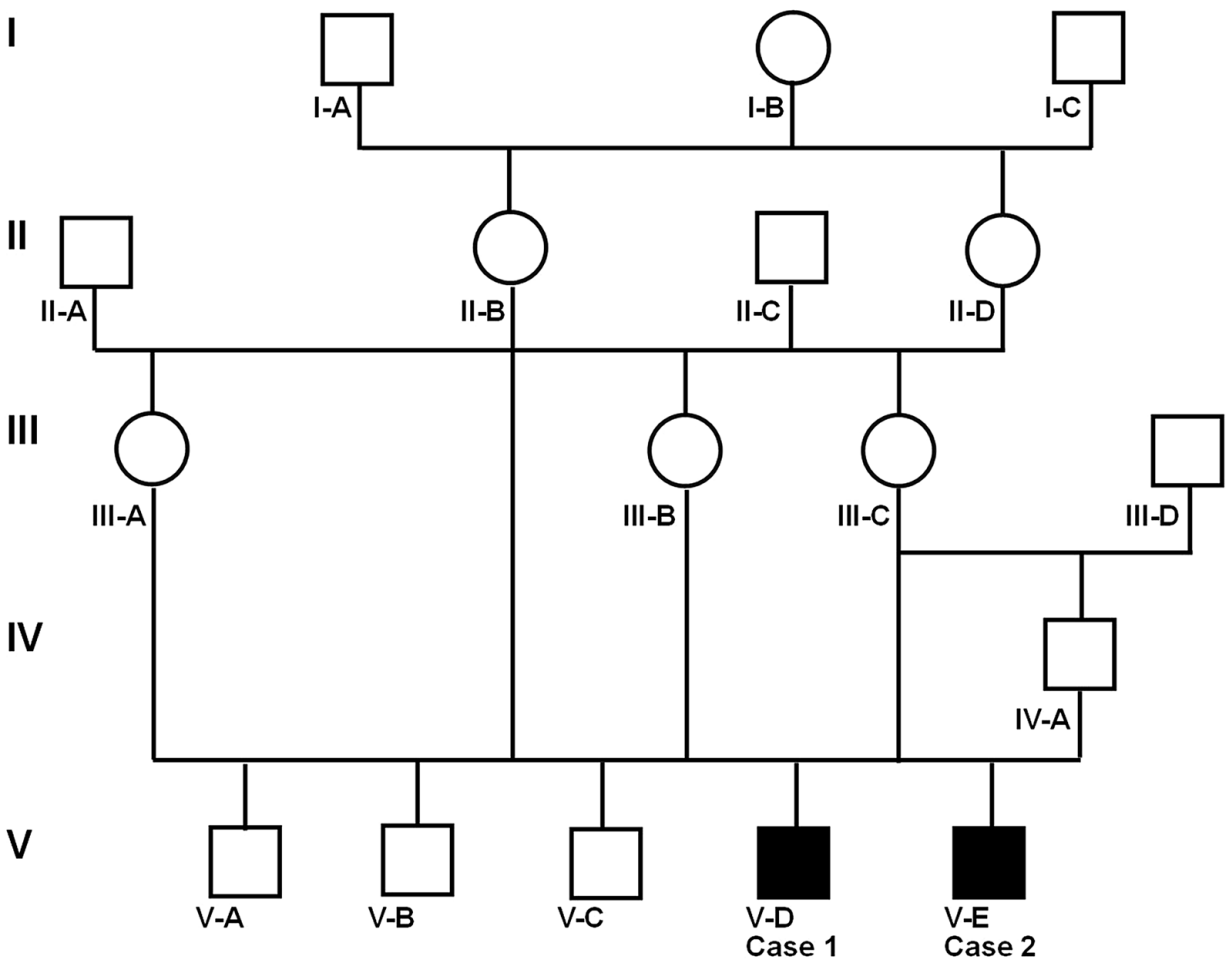
Familial relationship of the two PHA affected calves. Males are represented by *squares*, females by *circles*. *Full shading* designates affected

In April 2014, a 13-year-old Cika cow from a small farm counting 14 animals aborted in the seventh month of pregnancy. A male calf (V-D: case 1; Fig. 1), weighing 20 kg, was stillborn. After abortion the cow was euthanized due to exhaustion. In July 2014, a 14-year-old Cika cow from the same farm developed severe dystocia on the due date. A stillborn male calf (V-E: case 2; Fig. 1), weighing ~50 kg, was delivered by caesarean section. From previous mating with other bulls both cows (III-B and III-C; Fig. 1) had several normal offspring of both genders. Although the parentage of these two cases were not confirmed by DNA testing, examination of the pedigrees of both cases suggested that the two affected animals were inbred as both cows had been bred naturally with the same 2-year-old Cika bull (IV-A; Fig. 1). Furthermore this normally developed bull had been successfully mated to two other dams (II-B and III-A; Fig. 1), which were related to the dams of the affected calves, producing three normal male progeny. Pedigree analysis revealed inbreeding as case 1 was the result of an aunt-nephew mating (inbreeding coefficient 0.125) and case 2 of a dam-son mating (inbreeding coefficient 0.25). In addition, the dams were paternal half-sisters (relationship coefficient 0.25) and had a common maternal grandmother (I-B; Fig. 1). Taken together, the family history of both cases could be explained with transmission of monogenic autosomal recessive allele. Samples for DNA extraction were stored from both cases and their parents to allow a molecular genetic study in future.

**Fig. 2:**
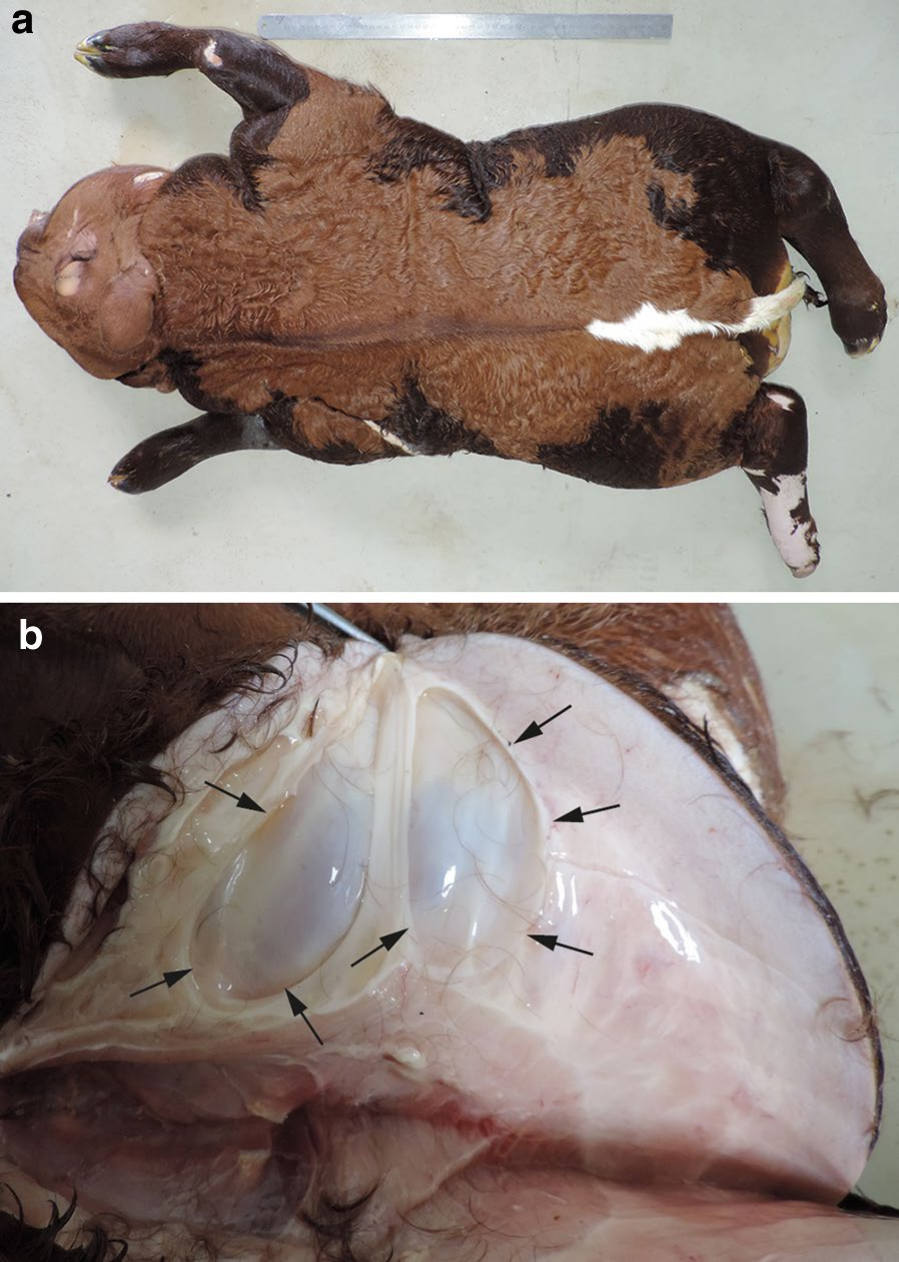
PHA in Cika cattle—gross pathology findings. **a** The body of the calf is severely deformed due to severe diffuse subcutaneous oedema. **b** The subcutis of the dorsal neck region is severely oedematous with multiple cysts (*arrows*). Case 2

**Fig. 3:**
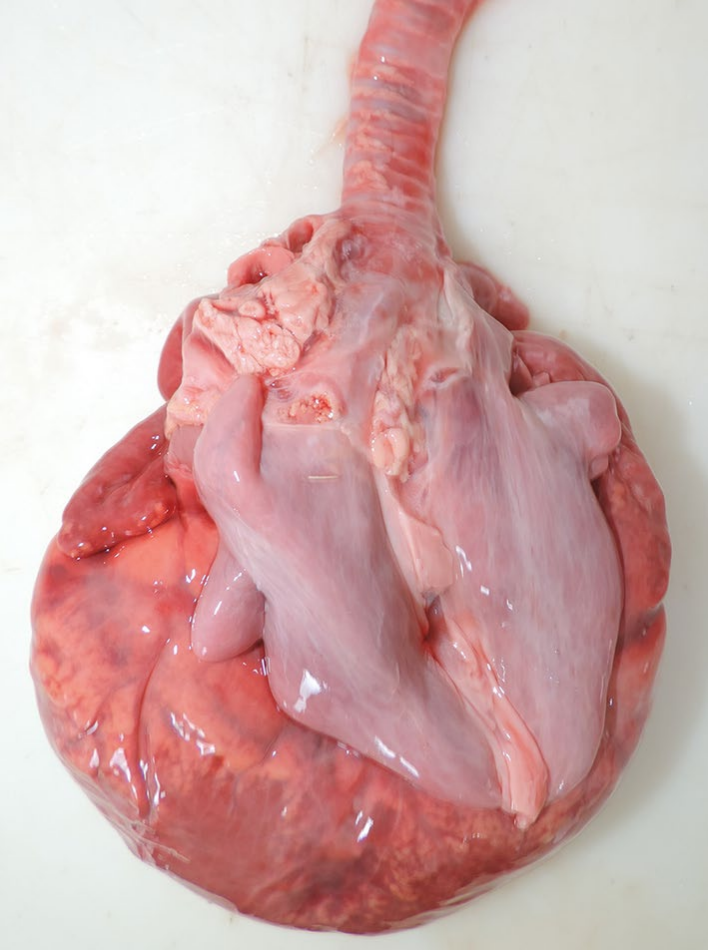
PHA in Cika cattle—gross findings of the lungs and the heart. Note the severe pulmonary hypoplasia and the enlarged, *rounded* heart due to marked dilatation of the heart chambers. Case 2

Gross lesions were similar in both affected calves. The bodies were severely deformed due to severe diffuse subcutaneous oedema with multiple cysts of various sizes, filled with serohemorrhagic fluid (Fig. 2). Hydrothorax, hydropericardium, ascites and an enlarged, rounded heart were also observed. The lungs were hypoplastic and poorly lobulated (Fig. 3). In both calves lymph nodes were not found, and in addition, case 1 was completely athymic.

**Fig. 4:**
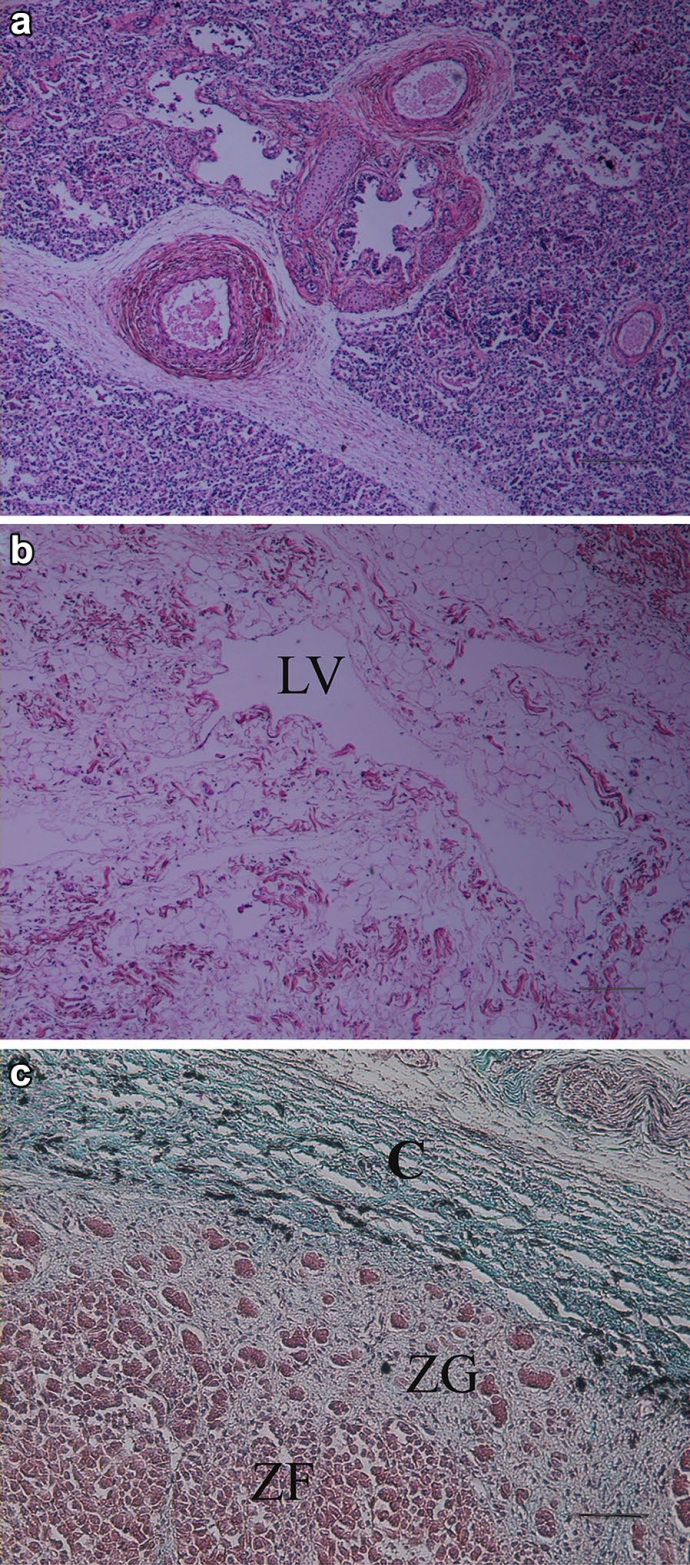
PHA in Cika cattle—histopathology of case 2. **a** The pulmonary parenchyma is hypoplastic and predominantly atelectatic. The interlobular interstitium and bronchial walls have to severe diffuse oedema. There is no bronchus associated lymphoid tissue. HE, *bar* = 500 µm. **b** The subcutis is severely oedematous with a severely dilated lymph vessel (*LV*). HE, *bar* = 500 µm. **c** The adrenal gland is hypoplastic with a thick fibrotic capsule (*C*) and cortical zona glomerulosa in which only small islands and rows of parenchymal cells are scattered (*ZG*). Zona fasciculata (*ZF*) is well preserved. Goldner’s trichrome stain, *bar* = 200 µm

Specimens of the lung of case 1 and several organs of case 2 were sampled for histopathology, fixed in 10 % neutral buffered formalin, processed by routine methods, embedded in paraffin, sectioned at 4 µm and stained with haematoxylin and eosin (HE). A sample of the adrenal gland was additionally stained with Goldner’s trichrome method. Histopathology of case 2 revealed severe diffuse oedema of the visceral pleura, interlobular connective tissue and bronchial walls. The pulmonary parenchyma was mostly atelectatic. No bronchus associated lymphoid tissue (BALT) was found. Thymic parenchyma had a normal structure, but the interstitium showed severe oedema and small multifocal infiltrates of eosinophilic granulocytes. The subcutis and skeletal muscles had severe diffuse oedemat with multifocal, severely dilated lymph vessels. The adrenal gland was hypoplastic, with a thickened, fibrotic capsule and cortical zona glomerulosa, in which small islands and rows of parenchymal cells were scattered (Fig. 4). The spleen was normally developed. The lung of case 1 was severely autolysed and not suited for histology.

## Conclusions

The gross lesions of both calves were consistent with previously reported cases of PHA in cattle [3–6]. The pedigree of the established Cika cattle family was compatible with monogenic autosomal recessive inheritance similar to other reported forms of familial PHA in cattle (OMIA 001562–9913) and HF in sheep (OMIA 000493–9940). A recent dominant acting de novo mutation which occurred in the sire could also be hypothesised as possible genetic cause. Alternatively, the observed malformations could represent phenocopies as the animals were born on the same farm in a common environment. However the number of calves is limited, submission of further cases is requested to clarify the aetiology. Nonetheless, this case report indicates the presence of an inherited form of PHA syndrome segregating in Slovenian Cika cattle, a breed were no inherited disorders have been recognized so far. The findings emphasize how inbreeding in cattle populations with small effective population sizes like Cika cattle [9] can lead to emergence of recessive defects. Therefore, inbreeding should be avoided as much as possible in populations of limited size. Furthermore, it is essential that domestic animal populations like the Cika breed are continuously monitored for the appearance of genetic defects, so that selection against deleterious alleles can be implemented as early as possible. The collected material of this study will be used in a future study aiming a developing a DNA test for carrier detection towards eradication of this lethal defect from the Cika breed. For Maine Anjou and Shorthorn cattle, a DNA test for PHA is offered also the causative missense mutation has not been reported so far [10].

Interestingly, in addition to HF and pulmonary hypoplasia, no lymph nodes were observed in our cases as has been reported previously in Maine-Anjou, Shorthorn and their crosses. Besides, one of the calves was also athymic, which was consistent with other reports [4, 6, 10]. However, in a Belted Galloway calf palatoschisis, superior and inferior brachygnathism, interventricular septal defect, dilatation of the pulmonary trunk and bilateral abdominal cryptorchidism were also described [5]. Similar malformations were also described in sheep; however, the cases were not diagnosed as PHA syndrome but as HF [8] and HF with anasarca [7]. Cases in sheep were associated with lymph node and lymph vessel agenesis, while the spleen, thymus and Peyer’s patches were normally developed [7, 8]. Neither the large ducts that convey lymph to the *cisterna chyli* nor the thoracic ducts were found. In addition to above quoted lesions, marked hypoplasia of the skeletal muscles and brachygnathia were also observed in affected sheep [8].

So far histopathological findings have been described in PHA affected Australian Dexter and Galloway calves [3, 5]. In a calf with hypoplastic lungs, severe oedema of the pleural surface, interalveolar spaces, peribronchial areas and bronchial submucosa, peribronchial fibrosis, increased cellularity of the alveolar septum, and pulmonary alveoli filled with abundant proteinaceous material resembling surfactant were described [3]. In a calf with aplastic lungs the thymus was present. The authors performed examination of a range of organs, but according to their description they did not notice lymph vessel abnormalities [3]. On the other side, histopathology of sheep affected with HF did not confirm the presence of lymph nodes and lymph vessels nor BALT [8], while the spleen, thymus and Peyer’s patches showed no significant changes [7, 8].

The gross lesions in all above mentioned defects in cattle and sheep are consistent with human HF. In the past, most of the human HF cases were due to erythroblastosis from Rhesus alloimmunization, while nowadays nonimmune HF prevails [1, 2]. Human nonimmune HF has most often been found in association with cardiovascular disorders (20.1 %), lymphatic dysplasia (15 %), haematological (9.3 %) and chromosomal (9.0 %) disorders [2]. While lymphatic dysplasia (i.e. lymphoedema and lymphangiectasia) is often recognized as a cause of HF in humans, it has not been reported as the cause of HF in animals yet. We speculate that severe oedema with lymphangiectasia that was found in the organs of PHA affected Cika calves, may indicate abnormalities in the development of the lymphatic system. Congenital lymphatic dysplasia may lead to reduced lymph flow, systemic lymphatic reflux, formation of ascites, pleural and pericardial effusions, increased interstitial fluid accumulation and development of nonimmune HF [11]. Furthermore, HF with excessive accumulation of the fluid in the thoracic cavity may result in pulmonary hypoplasia due to compression [12] and impaired lung maturation [13]. Mutations in genes, important for lymphatic development, are known as a possible cause of lymphatic dysplasia in humans. Mutations in more than 20 genes have been found to be responsible for developmental and/or functional defects affecting the lymphatic vessels in humans [14–16]. A genetic characterization of this bovine disorder has the potential to add a new locus to the list of genes of importance for lymphatic development or could confirm an association to the still unknown gene associated with PHA in other breeds of cattle.

## Abbreviations

BALT: bronchus associated lymphoid tissue
HE: haematoxylin and eosin
HF: hydrops foetalis
OMIA: online Mendelian inheritance in animals
OMIM: online Mendelian inheritance in man
PHA: pulmonary hypoplasia and anasarca
